# Comparison between logbook-reported and objectively-assessed physical activity and sedentary time in breast cancer patients: an agreement study

**DOI:** 10.1186/s13102-017-0072-2

**Published:** 2017-03-31

**Authors:** Anne-Sophie Mazzoni, Karin Nordin, Sveinung Berntsen, Ingrid Demmelmaier, Helena Igelström

**Affiliations:** 1 Department of Public Health and Caring Sciences, Section of lifestyle and rehabilitation in long term illness, Uppsala University, Box 564, BMC, Uppsala, S-75122 Sweden; 2 Department of Public Health, Sport and Nutrition, Faculty of Health and Sport Sciences, University of Agder, Box 422, Kristiansand, NO-4604 Norway

**Keywords:** Activity monitor, Breast neoplasms, Exercise, Measurement accuracy, Sedentary lifestyle

## Abstract

**Background:**

Increasing physical activity (PA) and decreasing sedentary time (ST) have important health effects among breast cancer patients, a growing population group. PA and sedentary behaviors are complex multi-dimensional behaviors and are challenging to monitor accurately. To date few studies have compared self-reports and objective measurement in assessing PA and ST in women undergoing breast cancer treatments. The aim of the present study was to compare self-reports and objective measures for assessing daily time spent in moderate-intensity physical activity (MPA), vigorous-intensity physical activity (VPA) and ST in women undergoing breast cancer treatments.

**Methods:**

Baseline data from 65 women with breast cancer scheduled to undergo adjuvant treatment was included. Daily time spent in MPA, VPA and ST was assessed by a study-specific logbook and the SenseWear Armband mini (SWA). The level of agreement between the two measurement methods was then determined by performing Bland-Altman plots with limits of agreements, and calculating Spearman’s rank correlation coefficients.

**Results:**

The mean difference between the logbook and SWA with limits of agreement was 14 (±102) minutes for MPA, 1 (±21) minute for VPA and −196 (±408) minutes for ST, respectively. The logbook reported an average of 34 and 50% higher values than the SWA for MPA and VPA, as well as an average of 27% lower values for ST (*P* < 0.05). The Spearman’s rank correlation coefficients showed that the differences between the methods increased as the average amount of time spent in PA and ST increased (*P* < 0.01).

**Conclusions:**

The results imply that the two measurement methods have limited agreement and cannot be used interchangeably.

## Background

Breast cancer is a major and growing public health concern, affecting millions of women worldwide [1]. Globally, breast cancer incidence rates have been constantly increasing the previous two decades, making breast cancer one of the leading causes of disability and cancer deaths in women [1]. Current evidence supports that participating in regular physical activity (PA; any bodily movement produced by skeletal muscles that increases energy expenditure above resting levels [2]), especially moderate-intensity physical activity (MPA) and vigorous-intensity physical activity (VPA), both during and after treatment, provides many health benefits for breast cancer patients [3, 4]. For example, PA is positively associated with improved health-related quality of life, psychosocial well-being and physical function [4], as well as reduced risk of cancer recurrence and mortality [3]. Emerging evidence also suggests that sedentary behaviors (any waking activity characterized by low levels of energy expenditure while in a sitting or reclining posture [5]) may have negative health effects for these populations, such as impaired health-related quality of life [6] and increased mortality risk [7].

PA and sedentary behaviors are important outcomes in cancer research studies focusing on these behaviors. Accurate monitoring of time spent in PA and sedentary time (ST) is thus essential for many reasons, inter alia, to understand the relationship between these behaviors and different health outcomes, to describe dose–response relationships and finally, to evaluate the impact and efficacy of public health interventions [8]. However, PA and sedentary behaviors are challenging to measure accurately because of their complex multi-dimensional nature [9]. The most commonly used measurement methods are self-reports such as questionnaires [9] and diaries (or logbooks) [10] due to their convenience (e.g. inexpensive, easily administered and used in different clinical purposes) [9, 10] and their ability to provide contextual information about different aspects of PA and sedentary behaviors (e.g. mode of activities, PA and sedentary behavior patterns) [9, 11]. However, they have limitations [10, 12] including an increased risk for recall and response bias due to social desirability and cognitive demands of recall [10, 13]. These issues may be even greater among breast cancer patients undergoing cancer treatments and experiencing disease and treatment-related symptoms such as cognitive impairments [14–16]. On the other hand, objective methods such as physical activity monitors like the SenseWear Armband (SWA) are now more widely used in breast cancer studies [17, 18] despite their cost [9], intrusiveness [12] and inability to provide contextual information about PA and sedentary behaviors (e.g. mode of activities, behavior patterns) [9]. They have the capacity to estimate the number and length of activity bouts and breaks in ST [9] as well as to remove the issues of recall and response bias [12, 13]. In fact, both self-reports and physical activity monitors are reported to have advantages and limitations [9, 10] and it appears that no “gold standard” exists for recording PA and ST in everyday life [9, 10] in breast cancer patients. However, given the importance of accurate monitoring of PA and ST among breast cancer populations, it is essential to determine the precision of self-reports compared with objective assessments, in order to know which measurement methods are the most appropriate. It is thus important to evaluate agreement between different commonly used methods [19]. However, few studies have compared self-reports and objective measurement in assessing PA [19–21] and ST [19, 20] in cancer populations. These studies have been conducted among different cancer populations and provide mixed results. Some studies have found acceptable [20] to good [21] agreement whereas the results of other studies have shown limited agreement [19, 20]. All these studies have included cancer patients post-treatment and only one has been conducted among breast cancer participants [20]. Thus, to date, the agreement between methods for assessing PA and ST in women with breast cancer undergoing cancer treatments has been poorly evaluated. The aim of the present study was to compare self-reports using a study-specific logbook and objective measures using the SWA for assessing daily time spent in MPA, VPA and ST in women with breast cancer undergoing cancer treatments.

## Methods

### Study participants

The present study is part of an on-going Swedish prospective study, the Physical Training and Cancer (Phys-Can) cohort study. The Phys-Can cohort study aims to monitor how the disease and treatment influence cancer patients’ physical fitness, mental well-being, quality of life and patterns of physical activity. In the Phys-Can cohort study, participants with newly diagnosed breast, colorectal or prostate cancer scheduled to undergo adjuvant treatment were consecutively recruited at three University hospitals in different regions of Sweden (Uppsala, Linköping and Lund). Patients with spread breast cancer (stadium IIIb-IV), cognitive dysfunction (e.g. dementia and serious mental illness), physical impairments or other diseases (e.g. cardiovascular and lung diseases) that can prevent engagement in physical activity were excluded.

Women with breast cancer (*n* = 84) who accepted to participate in the Phys-Can cohort study between September 2014 and April 2015 were included in the present study. The Phys-Can cohort study was approved by the Regional Ethical Review Board of Uppsala University (EPN D-number 2014/249) and all participants gave informed written consent.

### Procedure

Data assessments were conducted during cancer treatments, i.e. following the primary treatment (surgery) and before the participants started their adjuvant treatment. During a visit at the University hospital, each participant received a physical activity monitor and a study-specific logbook, and then was instructed on how to use these two instruments. After wearing the physical activity monitor 24 hours a day for seven consecutive days and filling out the logbook during the same period, the participants returned the two instruments by post in prepaid envelopes.

### Data collection and management methods

#### Physical activity monitor

The SenseWear Armband mini (BodyMedia Inc., Pittsburgh, PA, USA) was used in combination with the proprietary SenseWear Professional 8.1 software. The SWA was worn on the back of the upper triceps (right or left) as instructed by the manufacturer and assessed time spent in PA and ST by registering inputs from a three-axis accelerometer and a combination of heat sensors [22]. Data registered by the sensors was then integrated into proprietary algorithms to provide estimates of energy expenditure, assigning a Metabolic Equivalent value (MET - the ratio of the metabolic rate during exercise to a reference metabolic rate at rest [23]) to each minute the monitor was worn [22]. The proprietary SenseWear Professional Software provided SWA wear time as well as information about time spent in different MET levels, expressed in minutes [24]. Different generations of the SWA had previously been shown to be valid and reliable measurement methods in adult healthy populations [22, 25], as well as in adult cancer patients [26], compared to doubly labeled water [22] and indirect calorimetry [25, 26].

Collected data from the SWA was considered valid and was included in the analyses if the SWA was worn during at least 4 days [27], including 1 weekend day, with a wear time of at least 12 waking hours per day [28]. PA bouts considered for the analyses were defined as ten or more consecutive minutes [29] at the relevant intensity level, allowing interruptions of 1 or 2 min below threshold. Activities were excluded as a bout if they were interrupted by any ST or lower intensity levels of PA for more than 2 min [30]. Each bout of PA was classified as moderate or vigorous intensity, using cut-off values established by current international guidelines: 3.0–5.9 METs for MPA and ≥6.0 METs for VPA [31]. In order to identify and extract bouts of MPA and VPA of 10 min or more, visual analyses of the excel-files generated by the SenseWear Professional 8.1 software were made for each valid day from all the participants. Further, the participants’ daily time spent in MPA and VPA were calculated separately by summing minutes in a day where the count met the criterion for the relevant intensity. Additionally, all activities undertaken during daytime, with an energy expenditure ≤1.5 METs were classified as sedentary behaviors [5]. In order to delimit ST to only daytime, the time axes of each individual were visually analyzed and nighttime sleep was removed. Finally, using these delimitations, the participants’ daily ST was calculated and expressed in minutes.

#### Study-specific logbook

A logbook was developed for the Phys-Can cohort study, consisting of instruction pages and log pages for each day over 1 week. Participants made daily notes regarding ST as well as all PA that lasted 10 min or more, including mode, time and duration of the activities. The design of the study-specific logbook even enabled participants to rate their perceived exertion for each activity, using the commonly used Borg’s rating of perceived exertion 6 to 20 scale [32]. Similar logbooks have been previously used in several studies, involving other adult populations [33, 34].

Data from the logbook was used in the analyses only if the logbook was completed the same days as valid data from the SWA. The logbook of each participant was examined and days corresponding to the days of invalid data from the SWA were excluded from the analyses. As with the management of SWA-assessed data, the same MET classification as described above was used and a MET value was determined for each activity lasting 10 min or more using the Compendium of Physical Activities compiled by Ainsworth et al. [23]. Finally, using these delimitations, the participants’ daily time spent in MPA and VPA were calculated separately by summing minutes in a day where the count met the criterion for the relevant intensity. In addition, ST was determined as reported by the participants in the logbook.

### Data analysis

All data analyses were performed with the Statistical Package for the Social Sciences (SPSS v. 22.0, Inc. Chicago, Illinois, USA), and a two-tailed *P*-value of ≤0.05 was considered statistically significant.

Descriptive characteristics are presented as mean and standard deviation (SD) as well as frequencies and percentages (%). The average daily time spent in MPA, VPA and ST with the logbook and the SWA is presented at group level as mean with 95% confidence intervals (CI) ± SD. A Wilcoxon signed-rank test was performed to compare the differences (i.e. bias) between the two methods. Agreement between the logbook and the SWA was assessed as described by Bland and Altman [35]. Bland-Altman plots with values for the mean difference between the logbook and SWA as well as limits of agreements (mean difference ±1.96 SD) were created for MPA, VPA, and ST, and 95% CI of the mean differences were calculated. Spearman’s rank correlation coefficients (r) were then calculated to determine the association between the average and the difference in daily time spent in MPA, VPA and ST between the logbook and the SWA. The two methods were considered as having a good level of agreement and interchangeable if the mean difference and the range between the limits of agreement did not exceed 10, 5, and 60 min per day regarding MPA, VPA and ST respectively. These cut-off points were defined as a priori the limits of maximum acceptable differences based on research findings that indicate the importance of using measurement methods that are able to detect such clinical changes. Indeed, recent research indicates that an increase of 10 and 5 min of MPA and VPA per day respectively may lead to increased health benefits in the general population [36, 37]. Second, research regarding ST indicates that an increase of 60 min per day in ST is associated with increased health risk in the general population [38].

## Results

**Fig. 1:**
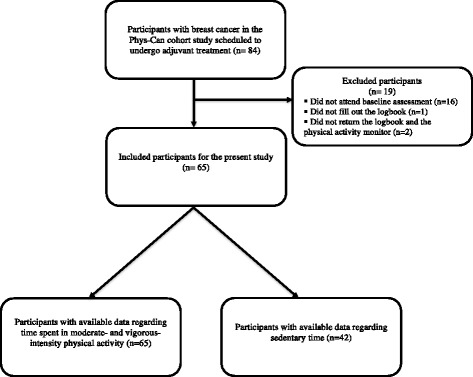
Overview of the study participants based on data from the Phys-Can cohort study

From the 84 breast cancer participants in the Phys-Can cohort study, 16 participants dropped out before attending baseline assessment. Three participants were then excluded because they had either not used or not returned the two measurement instruments. Finally, 65 participants were included in the present study. The characteristics of the 65 study participants are presented in Table 1. Regarding MPA and VPA, all 65 participants had available data. Regarding ST, only 42 participants had available data due to administrative failure (missing instruction for reporting ST in the first 23 logbooks) (Fig. 1).

**Table 1:** Characteristics of the study participants (*n* = 65)

Characteristic	Participants
*Age, year, mean (SD)*	58 (11)
*Geographical location, n (%)*	
Malmö/Lund	14 (22)
Linköping	24 (37)
Uppsala	27 (41)
*Marital/Partner status, n (%)* ^*a*^	
Single	12 (19)
Unmarried/married cohabitation	46 (74)
Living Apart Together	4 (7)
*Occupation, n (%)* ^*a*^	
Working	18 (29)
On sick leave	20 (32)
Retired	22 (36)
Other	2 (3)
*Level of education, n (%)* ^*b*^	
Elementary school	5 (8)
High school	25 (41)
College/university	31 (51)
*Scheduled adjuvant treatment regime, n (%)* ^*c*^	
Chemotherapy therapy	19 (32)
Radiation therapy	15 (25)
Hormonal therapy	19 (32)
Combination	7 (11)
*Self-reported comorbidity, n (%)* ^*a*^	
None	31 (50)
One or more	31 (50)

*Abbreviations*: *SD* standard deviation

Missing data from ^a^ three participants, ^b^ four participants and ^c^ five participants

### Time spent in MPA, VPA and ST

As shown in Table 2, the average daily time spent in MPA (essentially walking and bicycling activities according to the logbook) for the entire group was 55 and 41 min according to the logbook and the SWA, respectively. The entire group spent an average of 3 and 2 min per day in VPA, according to the logbook and the SWA, respectively. The average daily time spent in both MPA and VPA was significantly higher for the logbook compared with the SWA (34%, *P* < 0.01 and 50%, *P* = 0.014, respectively).

**Table 2:** Comparison of the study- specific logbook and the SenseWear Armband mini - monitored time spent in sedentary behaviour, moderate- and vigorous- intensity physical activity

Activity intensity^a^	Daily minutes		
	Logbook	SWA	*P*-value
Moderate	55 (50, 60) ± 54	41(37, 45) ± 38	<0.01
Vigorous	3 (2, 4) ± 12	2 (1, 3) ± 7	0.014
Sedentary	517 (494, 540) ± 185	713 (697, 729) ± 128	<0.01

*Abbreviations*: *SWA* SenseWear Armband mini

^a^Activity intensity (METs) cut-off points = sedentary (≤1.5 METs), moderate (3.0–5.9 METs), vigorous (≥6.0 METs)

Values are presented as mean minutes per day (95% confidence intervals) ± standard deviation

Regarding sedentary behaviors, the average daily ST for the entire group was significantly lower for the logbook compared with the SWA (−27%, *P* < 0.01). The entire group spent an average of 517 and 713 min per day in sedentary behaviors, according to the logbook and the SWA, respectively.

### Agreement between methods assessing time spent in MPA, VPA and ST

**Fig. 2:**
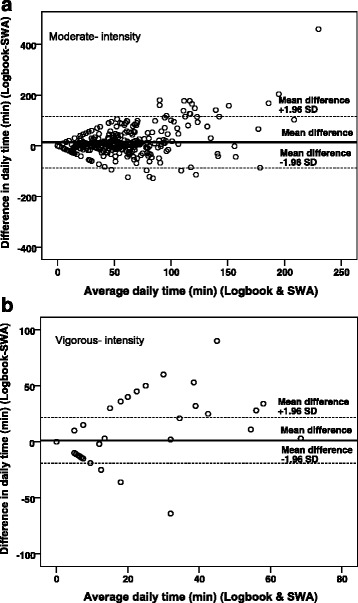
**a** Bland-Altman *plot* of the difference in daily time (*min*) spent in moderate- intensity physical activity between the logbook and the SenseWear Armband mini (*SWA*) against the average of values measured by the two methods with 95% limits of agreement. **b** Bland-Altman *plot* of the difference in daily time (*min*) spent in vigorous- intensity physical activity between the logbook and the SenseWear Armband mini (*SWA*) against the average of values measured by the two methods with 95% limits of agreement. Each *point* represents a pair of measurement (*logbook and SWA*) obtained from the 65 participants, who had valid data from four to seven days, totalling 409 comparisons. The *thick lines* indicate the mean difference and the *broken lines* indicate the limits of agreement

Figure 2a shows a Bland-Altman plot comparing daily time spent in MPA between the logbook and the SWA. The mean difference between the logbook and the SWA was 14 min per day (95% CI [9, 19]) with limits of agreement of ±102 min. Differences between the logbook and the SWA increased as the average minutes per day spent in MPA increased (*r* = 0.3, *P* < 0.01).

The Bland-Altman plot for daily time spent in VPA from the logbook and the SWA showed a mean difference of 1 min per day (95% CI [0.3, 2]) with limits of agreement of ±21 min (Fig. 2b). Differences between the logbook and the SWA also increased as the average minutes per day spent in VPA increased (*r* = 0.5, *P* < 0.01).

**Fig. 3:**
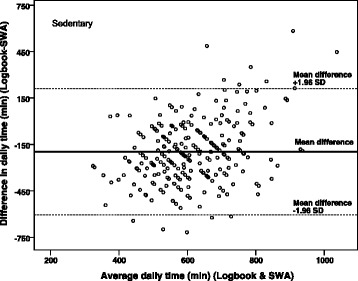
Bland-Altman *plot* of the difference in daily sedentary time (*min*) between the logbook and the SenseWear Armband mini (*SWA*) against the average of values measured by the two methods with 95% limits of agreement. Each *point* represents a pair of measurement (*logbook and SWA*) obtained from the 42 participants, who had valid data from four to seven days, totalling 253 comparisons. The *thick line* indicates the mean difference and the *broken lines* indicate the limits of agreement

Figure 3 shows a Bland-Altman plot of daily ST as measured by the two methods. The mean difference between the logbook and the SWA was −196 min per day (95% CI [−221, −170]) with the limits of agreement of ±408 min. Differences between the logbook and the SWA increased as the average minutes per day spent in sedentary behaviors increased (*r* = 0.4, *P* < 0.01).

## Discussion

The results of the present study showed significant mean differences, wide limits of agreement, and significant positive correlations between the logbook and the SWA regarding the average and the difference in daily time spent in MPA, VPA and ST. Thus, the results indicated limited agreement between the logbook and the SWA, suggesting that the two methods cannot be used interchangeably for obtaining accurate measures in daily time spent in PA and ST among breast cancer patients undergoing cancer treatments.

Similar findings have been obtained in previous studies. A study conducted by Johnson-Kozlow et al. [20], including 159 breast cancer patients post-treatment reported limited agreement between one self-report method and an accelerometer in measuring time spent in MPA, VPA and total PA as well as poor agreement in measuring ST. A multicenter study [19] with 176 colon cancer patients reported limited agreement between two self-report methods and an accelerometer, regarding MPA, VPA and ST. In contrast, Jovanovic et al. [21] reported high agreement between a daily diary and an accelerometer in measuring time spent in PA among 23 women with endometrial cancer. Two of the last mentioned studies included other cancer diagnosis compared to the present study [19, 21] and all were conducted post-treatment, making it difficult to draw any certain conclusions.

The results in the present study indicated that the participants tended to overestimate PA (at both moderate and vigorous intensity) and underestimate ST when using the logbook. This is also in line with both the study by Johnson-Kozlow et al. [20] and the multicenter study by Boyle at al [19]. The observed overestimation of time spent in MPA, VPA and the underestimation of ST may be explained by some limitations of the logbook, i.e. over- and under-reporting due to the failure to recall frequency and duration of these behaviors correctly, a common limitation of self-reports. Indeed, self-report instruments have difficulties in establishing the true frequency, duration and intensity of the activities [10] and have an increased risk for recall and response bias due to the cognitive demands of recall and social desirability [10, 13]. The risk of recall bias may be higher among breast cancer patients due to cognitive impairments that can appear during treatments [14–16]. This can result in altered cognitive functions such as reduced memory [14–16] and decreased concentration /attention [15], which in turn may affect the quality of self-report of PA and ST among these populations. However, the SWA also may have contributed to the differences in daily time spent in PA and ST between the two measurement methods found in the present study. Although validity studies showed that the SWA is valid compared to indirect calorimetry and doubly labeled water in adult healthy populations [22, 25] and in adult cancer patients [26], the same studies showed that the SWA tends to underestimate total energy expenditure during moderate- and vigorous-intensity activities such as walking and ergocycling when compared to indirect calorimetry [26]. Those studies also showed that the SWA tends to overestimate total energy expenditure during physical activities of low intensity [22, 25]. This suggests that the SWA may have underestimated and misclassified physical activities such as cycling and walking exercises, which were the two most common forms of self-reported physical activities among the study participants according to the logbook. It also suggests that the SWA may have misclassified sedentary activities due to its difficulties to discriminate activities of low intensity (e.g. standing very stationary) from sedentary behaviors.

What is an acceptable level of agreement between two measurement methods is a clinical judgment based on whether the differences are small enough to avoid problems with clinical interpretation [39]. The criteria used in the present study suggested that the study-specific logbook might not be sensitive enough to detect such changes in time spent in MPA and VPA as well as ST. The results also suggest a larger level of agreement between the SWA and the logbook in assessing daily VPA compared to when assessing MPA and ST. However, it is important to highlight that the logbook gave statistically higher estimates of VPA, and that these differences increased as the amount of VPA increased, which in turn confirms the limited agreement between the two measurement methods in assessing VPA. It is also worth noting that few vigorous activities were performed by the participants as indicated by both the logbook and the SWA. Thus, the observed agreement between the two methods in measuring time spent in VPA must be carefully interpreted and needs to be further evaluated. Further, several studies [21, 34, 40] made interesting observations, suggesting that the logbook may instead be an appropriate instrument to complement the SWA, due to its ability to provide detailed and contextual information about different aspects of PA and sedentary behaviors (e.g. settings, mode of activities and self-perceived exertion). According to these studies, a combination of both objective and subjective monitoring may be beneficial to obtain detailed descriptions of PA and sedentary behavior patterns among the studied population, and may be more likely to yield reliable measurements and understanding of these behaviors. Evidently, the choice of measurement methods depends mainly on the purpose for assessing PA and ST, and the decision of selecting the most appropriate method (or a combination of several methods) should be made on a case-by-case basis [9–11].

The present study has some limitations and the results should therefore be interpreted accordingly. First, the relatively small sample size and its homogeneity could have an impact on the results. Recommendations about sample sizes for agreement studies are few [41] and vary widely, ranging from at least 32 participants [42] to over 100 participants [43]. This makes naturally difficult to draw any certain conclusion about optimal sample sizes. Further, most of the study participants were well-educated and all were volunteers to participate in the study. Thus, these limitations may affect the representativeness of the study sample and limit the generalizability of the results to larger breast cancer populations. However, the data was collected at three different hospitals in different regions of Sweden, allowing a reduction of possible influences of e.g. community characteristics. This, in turn, may even contribute to give a more representative sample of a greater breast cancer population. Second, since there is no gold standard for recording PA and ST in everyday life [9, 10], the SWA was used as a comparison measure despite its limitations mentioned above. Third, the great variability (SDs) of daily time spent in PA and ST may also impact on the results. As the Spearman’s rank correlation coefficients show, differences between the logbook and the SWA increased as the average minutes spent in PA and ST increased, suggesting that the days the participants were the most physical active or the most sedentary, were also the days where the level of agreement between the SWA and the logbook was poorest and vice versa. PA levels vary from day to day and several sources explain those variations such as weekend/weekday [44] and seasons [45], implying that the observed level of agreement may differ depending on 1) which season of the year the data is collected and 2) the choice of valid days for the analysis. However, to limit these methodological issues, the data was collected during different seasons (i.e. fall, winter and spring) for the entire group, and the analysis encompassed at least 4 days, including 1 weekend day for each participant. Another limitation is the absence of guideline recommendations for determining acceptable bias when comparing subjective and objective measurements that record time spent in PA and ST. Due to this limitation, specific criteria were developed for the present study, but further research on this topic is necessary in order to identify cut-off points that can be used in a systematic manner for comparisons between studies. Furthermore, assessing PA and ST with a physical activity monitor also includes some technical and practical considerations [9], which may have an impact on the results [30]. For example, there is no consensus regarding data management and processing [9]. This lack of standardized data analysis strategies makes it difficult to choose the most appropriate way to manage SWA-data, and limits the possibility to compare studies. However, in order to limit these methodological issues, recent published recommendations [27, 28, 30] and guidelines [46, 47] were followed to analyze SWA-data in the present study (such as minimum number of valid days of monitoring required, minimum wear time, criteria for valid PA bouts). Additionally, the SWA was used in combination with proprietary software with proprietary algorithms to estimate data regarding time spent in PA and ST. This enabled a structured and systematic way of data management, allowing homogeneous interpretations of the results as well as comparisons between studies [48].

## Conclusions

In summary, the present study is the first to investigate agreement between self-reports and objective assessments with participants undergoing breast cancer treatments. The study-specific logbook has limited agreement with the SWA and cannot be used interchangeably with the SWA when assessing daily time spent in MPA, VPA and ST among breast cancer patients. However, the selection of method to assess PA and ST should be closely linked to clinical and research purposes. When appropriate, the logbook may be a useful complement to the objective measures due to its ability to provide detailed and contextual information about different aspects of PA and sedentary behaviors.

## Abbreviations

MET: Metabolic equivalent
MPA: Moderate-intensity physical activity
PA: Physical activity
Phys-Can: Physical Training and Cancer
ST: Sedentary time
SWA: SenseWear Armband mini
VPA: Vigorous-intensity physical activity

## References

[1] Fitzmaurice C, Dicker D, Pain A, Hamavid H, Moradi-Lakeh M, Global Burden of Disease Cancer Collaboration (2015). The Global Burden of Cancer 2013. JAMA Oncol.

[2] Thompson PD, Buchner D, Pina IL, Balady GJ, Williams MA, Marcus BH (2003). Exercise and physical activity in the prevention and treatment of atherosclerotic cardiovascular disease: a statement from the Council on Clinical Cardiology (Subcommittee on Exercise, Rehabilitation, and Prevention) and the Council on Nutrition, Physical Activity, and Metabolism (Subcommittee on Physical Activity). Circulation.

[3] Ibrahim EM, Al-Homaidh A (2011). Physical activity and survival after breast cancer diagnosis: meta-analysis of published studies. Med Oncol.

[4] Schmitz KH, Courneya KS, Matthews C, Demark-Wahnefried W, Galvão DA, Pinto BM (2010). American College of Sports Medicine roundtable on exercise guidelines for cancer survivors. Med Sci Sports Exerc.

[5] Sedentary Behaviour Research Network (2012). Letter to the editor: standardized Use of the terms “sedentary” and “sedentary behaviours”. Appl Physiol Nutr Metab.

[6] George SM, Alfano CM, Groves J, Karabulut Z, Haman KL, Murphy BA, Matthews CE (2014). Objectively measured sedentary time is related to quality of life among cancer survivors. PLoS One.

[7] George SM, Smith AW, Alfano CM, Bowles HR, Irwin ML, Mctiernan A (2013). The association between television watching time and all-cause mortality after breast cancer. J Cancer Surviv.

[8] Bauman A, Phongsavan P, Schoeppe S, Owen N (2006). Physical activity measurement—a primer for health promotion. Promot Educ.

[9] Broderick JM, Ryan J, O’Donnell DM, Hussey J (2014). A guide to assessing physical activity using accelerometry in cancer patients. Support Care Cancer.

[10] Warren JM, Ekelund U, Besson H, Mezzani A, Geladas N, Vanhees L (2010). Assessment of physical activity—a review of methodologies with reference to epidemiological research: a report of the exercise physiology section of the European Association of Cardiovascular Prevention and Rehabilitation. Eur J Cardiovasc Prev Rehabil.

[11] Ainsworth B, Cahalin L, Buman M, Ross R (2015). The current state of physical activity assessment tools. Prog Cardiovasc Dis.

[12] Prince SA, Adamo KB, Hamel ME, Hardt J, Connor Gorber S, Tremblay M (2008). A comparison of direct versus self-report measures for assessing physical activity in adults: a systematic review. Int J Behav Nutr Phys Act.

[13] Bassett DR, Troiano RP, McClain JJ, Wolff DL (2015). Accelerometer-based physical activity: total volume per day and standardized measures. Med Sci Sports Exerc.

[14] Ahles TA, Saykin AJ, McDonald BC, Furstenberg CT, Cole BF, Hanscom BS (2008). Cognitive function in breast cancer patients prior to adjuvant treatment. Breast Cancer Res Treat.

[15] Wefel JS, Lenzi R, Theriault RL, Davis RN, Meyers CA (2004). The cognitive sequelae of standard-dose adjuvant chemotherapy in women with breast carcinoma: results of a prospective, randomized, longitudinal trial. Cancer.

[16] Wefel JS, Lenzi R, Theriault R, Buzdar AU, Cruickshank S, Meyers CA (2004). ‘Chemobrain’ in breast carcinoma?: a prologue. Cancer.

[17] Yee J, Davis GM, Beith JM, Wilcken N, Currow D, Emery J (2014). Physical activity and fitness in women with metastatic breast cancer. J Cancer Surviv.

[18] Villarini A, Pasanisi P, Traina A, Mano MP, Bonanni B, Panico S (2012). Lifestyle and breast cancer recurrences: the DIANA-5 trial. Tumori.

[19] Boyle T, Lynch BM, Courneya KS, Vallance JK (2015). Agreement between accelerometer-assessed and self-reported physical activity and sedentary time in colon cancer survivors. Support Care Cancer.

[20] Johnson-Kozlow M, Sallis JF, Gilpin EA, Rock CL, Pierce JP (2006). Comparative validation of the IPAQ and the 7-Day PAR among women diagnosed with breast cancer. Int J Behav Nutr Phys Act.

[21] Jovanovic JL, Hughes DC, Baum GP, Carmack C, Greisinger AJ, Basen-Engquist K (2011). Accelerometry and self-report in sedentary populations. Am J Health Behav.

[22] Johannsen DL, Calabro MA, Stewart J, Franke W, Rood JC, Welk GJ (2010). Accuracy of armband monitors for measuring daily energy expenditure in healthy adults. Med Sci Sports Exerc.

[23] Ainsworth BE, Haskell WL, Herrmann SD, Meckes N, Bassett DR, Tudor-Locke C (2011). 2011 Compendium of Physical Activities: a second update of codes and MET values. Med Sci Sports Exerc.

[24] SenseWear. The New Software Version 8.1. Medshop. 2014. http://www.medshop.pl/uploads/files/SenseWear%208.1%20Features%20Functions-en-pl.pdf. Accessed 22 Mar 2015.

[25] Jakicic JM, Marcus M, Gallagher KI, Randall C, Thomas E, Goss FL (2004). Evaluation of the SenseWear Pro Armband to assess energy expenditure during exercise. Med Sci Sports Exerc.

[26] Cereda E, Turrini M, Ciapanna D, Marbello L, Pietrobelli A, Corradi E (2007). Assessing energy expenditure in cancer patients: a pilot validation of a new wearable device. JPEN J Parenter Enteral Nutr.

[27] Trost SG, McIver KL, Pate RR (2005). Conducting accelerometer-based activity assessments in field-based research. Med Sci Sports Exerc.

[28] Herrmann SD, Barreira TV, Kang M, Ainsworth BE (2014). Impact of accelerometer wear time on physical activity data: a NHANES semisimulation data approach. Br J Sports Med.

[29] Buffart LM, Galvão DA, Brug J, Chinapaw MJ, Newton RU (2014). Evidence-based physical activity guidelines for cancer survivors: current guidelines, knowledge gaps and future research directions. Cancer Treat Rev.

[30] Mâsse LC, Fuemmeler BF, Anderson CB, Matthews CE, Trost SG, Catellier DJ (2005). Accelerometer data reduction: a comparison of four reduction algorithms on select outcome variables. Med Sci Sports Exerc.

[31] Physical Activity Guidelines Advisory Committee (2008). Physical Activity Guidelines Advisory Committee Report, 2008.

[32] Borg GA (1974). Perceived exertion. Exerc Sport Sci Rev.

[33] Igelström H, Emtner M, Lindberg E, Asenlöf P (2013). Physical activity and sedentary time in persons with obstructive sleep apnea and overweight enrolled in a randomized controlled trial for enhanced physical activity and healthy eating. Sleep Breath.

[34] Igelström H, Emtner M, Lindberg E, Asenlöf P (2013). Level of agreement between methods for measuring moderate to vigorous physical activity and sedentary time in people with obstructive sleep apnea and obesity. Phys Ther.

[35] Bland JM, Altman DG (1999). Measuring agreement in method comparison studies. Stat Methods Med Res.

[36] Lee DC, Pate RR, Lavie CJ, Sui X, Church TS, Blair SN (2014). Leisure-time running reduces all-cause and cardiovascular mortality risk. J Am Coll Cardiol.

[37] Murakami H, Tripette J, Kawakami R, Miyachi M (2015). “Add 10 min for your health”: the new Japanese recommendation for physical activity based on dose–response analysis. J Am Coll Cardiol.

[38] Dunstan DW, Salmon J, Owen N (2005). Associations of TV viewing and physical activity with the metabolic syndrome in Australian adults. Diabetologia.

[39] Bland JM, Altman DG (2003). Applying the right statistics: analyses of measurement studies. Ultrasound Obstet Gynecol.

[40] Clemes SA, David BM, Zhao Y, Han X, Brown W (2012). Validity of two self-report measures of sitting time. J Phys Act Health.

[41] Kottner J, Audigé L, Brorson S, Donner A, Gajewski BJ, Hróbjartsson A (2011). Guidelines for Reporting Reliability and Agreement Studies (GRRAS) were proposed. J Clin Epidemiol.

[42] Liao JJ (2010). Sample size calculation for an agreement study. Pharm Stat.

[43] Mcalinden C, Khadka J, Pesudovs K (2011). Statistical methods for conducting agreement (comparison of clinical tests) and precision (repeatability or reproducibility) studies in optometry and ophthalmology. Ophthalmic Physiol Opt.

[44] Matthews CE, Ainsworth BE, Thompson RW, Bassett DR (2002). Sources of variance in daily physical activity levels as measured by an accelerometer. Med Sci Sports Exerc.

[45] Buchowski MS, Choi L, Majchrzak KM, Acra S, Mathews CE, Chen KY (2009). Seasonal changes in amount and patterns of physical activity in women. J Phys Act Health.

[46] Matthews CE, Hagströmer M, Pober DM, Bowles HR (2012). Best practices for using physical activity monitors in population-based research. Med Sci Sports Exerc.

[47] Ward DS, Evenson KR, Vaughn A, Rodgers AB, Troiano RP (2005). Accelerometer use in physical activity: best practices and research recommendations. Med Sci Sports Exerc.

[48] Bassett DR, John D (2010). Use of pedometers and accelerometers in clinical populations: validity and reliability issues. Phys Ther Rev.

